# Using an electronic activity monitor system as an intervention modality: A systematic review

**DOI:** 10.1186/s12889-015-1947-3

**Published:** 2015-06-24

**Authors:** Zakkoyya H. Lewis, Elizabeth J. Lyons, Jessica M. Jarvis, Jacques Baillargeon

**Affiliations:** Division of Rehabilitation Sciences, University of Texas Medical Branch (UTMB), 301 University Blvd, Galveston, TX USA; Department of Preventive Medicine & Community Health, UTMB, Galveston, TX USA; Department of Nutrition and Metabolism, UTMB, Galveston, TX USA; Center for Interdisciplinary Research in Women’s Health, UTMB, Galveston, TX USA; Division of Epidemiology and Outcomes, Correctional Managed Care, UTMB, Galveston, TX USA

**Keywords:** Activity monitor, Wearable technology, Activity tracker, Physical activity, Weight, Obesity, Technology, Self-monitoring, Systematic review

## Abstract

**Background:**

Obesity is a growing global health concern that may lead to cardiovascular disease, type II diabetes, and cancer. Several systematic reviews have shown that technology is successful in combating obesity through increased physical activity, but there is no known review on interventions that use an electronic activity monitor system (EAMS). EAMSs are defined as a wearable device that objectively measures lifestyle physical activity and can provide feedback, beyond the display of basic activity count information, via the monitor display or through a partnering application to elicit continual self-monitoring of activity behavior. These devices improve upon standard pedometers because they have the ability to provide visual feedback on activity progression, verbal encouragement, and social comparison. This systematic review aimed to synthesize the efficacy and feasibility results of EAMSs within published physical activity interventions.

**Methods:**

Electronic databases and journal references were searched for relevant articles. Data sources included CINAHL, Cochrane CENTRAL, Medline Ovid, PsycINFO, and clinicaltrials.gov. Out of the 1,574 retrieved, 11 articles met the inclusion criteria. These articles were reviewed for quality and content based on a risk of bias tool and intervention components.

**Results:**

Most articles were determined to be of medium quality while two were of low quality, and one of high quality. Significant pre-post improvements in the EAMS group were found in five of nine studies for physical activity and in four of five studies for weight. One found a significant increase in physical activity and two studies found significant weight loss in the intervention group compared with the comparator group. The EAMS interventions appear to be feasible with most studies reporting continual wear of the device during waking hours and a higher retention rate of participants in the EAMS groups.

**Conclusion:**

These studies provide preliminary evidence suggesting that EAMS can increase physical activity and decrease weight significantly, but their efficacy compared to other interventions has not yet been demonstrated. More high-quality randomized controlled trials are needed to evaluate the overall effect of EAMS, examine which EAMS features are most effective, and determine which populations are most receptive to an EAMS.

**Electronic supplementary material:**

The online version of this article (doi:10.1186/s12889-015-1947-3) contains supplementary material, which is available to authorized users.

## Background

Obesity is a rising pandemic. The World Health Organization estimates that 11 % of men and 15 % of women world-wide are classified as obese [1]. Approximately 21 % and 35 % of the population older than 20 years of age are obese in Western Europe and in the US, respectively [2, 3]. Obesity is a preventable condition that can lead to heart disease, stroke, type II diabetes and certain cancers and incurs billions of dollars in medical expenses [4]. In view of this, there are tremendous health and economic incentives to control obesity.

Approximately 23 % of adults world-wide do not meet the recommended levels of physical activity [1]. These rates are higher in high-income countries like the United States and countries in Western Europe [1]. Physical activity plays a fundamental role in controlling obesity. Jakicic et al. reported that 150 minutes of PA a week can result in up to 3 kg of weight loss over time [5]. Even in interventions that focus on eating, PA can improve weight losses and ameliorate weight regain and thus is a critical component of interventions [6].

Face-to-face behavioral lifestyle interventions are conducted to positively change PA behavior in order to control weight [7]. These interventions do so by delivering behavior change techniques (BCTs). Commonly used BCTs include shaping knowledge, social support, goal setting, and self-monitoring [8]. Face-to-face interventions that incorporate these BCTs result in an increase in physical activity of approximately 81 to 982.4 kcal per week [9] and approximately 8 to 10 kg of weight loss [8]. The magnitude of improvement varies by participant characteristics, such as age [9], but standard behavioral interventions have demonstrated effectiveness in improving behaviors and weight outcomes [8]. Unfortunately, these interventions are costly and require professional expertise in delivering BCTs [8]. Fundamental BCTs present in these interventions include self-monitoring, feedback, and social support [7, 10–13]. Self-monitoring is among the most effective techniques within PA interventions [14]. In recent years, new technologies have been used for self-monitoring as well as delivering other BCTs [11].

Technology is used in several forms (i.e., internet, mobile phones, activity monitors) [12, 15]. Technology-based interventions began by utilizing websites, but as technology advanced interventions have progressed to using more sophisticated media such as mobile monitoring [16]. Technology is favored in interventions because devices are typically portable, allow for continual self-monitoring, cost-effective, convenient, accessible, and give the user a sense of control [7, 12, 17]. Furthermore, there is evidence that technology is an effective mode of delivery for an intervention that can result in weight loss improvement and PA promotion, independent of face-to-face contacts [7, 18–21]. Khaylis et al. [12] reported the five vital components incorporated in technology-based interventions: self-monitoring, counselor feedback, social support, structure and principles of behavior change, and an individually tailored program [12]. These components were established from a review of widely used technologies. One new technology not exclusively reviewed is an electronic activity monitor system (EAMS). EAMSs are commercially popular and have been evaluated in individual studies, but there is no known systematic investigation of their efficacy [22].

For the purposes of this review an EAMS will be defined as *a wearable device that objectively measures lifestyle PA and can provide feedback, beyond the display of basic activity count information, via the monitor display or through a partnering application to elicit continual self-monitoring of activity behavior*. This definition eliminates pedometers that provide only very basic activity count feedback and accelerometers that do not give automated feedback to the wearer. Along with activity count information, an EAMS has the ability to provide visual feedback on activity progression, verbal encouragement, and social comparison via a mobile device or personal computer. Some of the systems are commercially available, while others were developed by a research team. Commercially available EAMSs are growing in popularity with an estimated 3.3 million units sold in 2014 [23] generating about $238 million in sales [24]. An EAMS is equipped with several fundamental BCTs related to change in PA behavior defined by Michie et al. [11]. These include techniques such as goal setting, review of behavioral goals, and social support [25]. EAMSs also have the ability to incorporate most of the vital components described by Khaylis et al. [12, 25].

Increasing interest among researchers, practitioners, and individuals in these monitors inspires a systematic investigation into their feasibility and efficacy [22, 25–27]. EAMS have the potential to deliver multiple BCTs, potentially in ways that are as or more effective than traditional methods [25]. Due to the possible benefits from utilizing EAMSs in an intervention, there is a need to accumulate existing evidence to guide and inform future research. The purpose of this review was to synthesize the available information on efficacy and feasibility of EAMSs as a modality within a PA intervention for adults.

## Methods

This review was conducted in accordance with the Preferred Reporting Items for Systematic Reviews and Meta-analysis (PRISMA) statement [28].

### Search Strategy

The search strategy was developed with the guidance of a trained reference librarian. Articles were collected from CINAHL, Cochrane CENTRAL, Medline Ovid, and PsycINFO online databases. Broad search terms were used: activity monitor, adults and PA. Related terms and phrases were also used. Adults were specified because child monitors are often substantially different from those used among adults. The Medline Ovid search strategy is shown in Table 1. Other articles were retrieved from clinicaltrials.gov and reference searching.

**Table 1:** Medline Ovid search strategy

#	Searches	Results	#	Searches	Results
1	electronic monitor*.mp.	867	11	exercis*.mp. [mp = title, abstract, original title, name of substance word, subject heading word, keyword heading word, protocol supplementary concept word, rare disease supplementary concept word, unique identifier]	258198
2	electronic track*.mp. [mp = title, abstract, original title, name of substance word, subject heading word, keyword heading word, protocol supplementary concept word, rare disease supplementary concept word, unique identifier]	67	12	9 or 10 or 11	324327
3	electronic activ* monitor*.mp. [mp = title, abstract, original title, name of substance word, subject heading word, keyword heading word, protocol supplementary concept word, rare disease supplementary concept word, unique identifier]	29	13	Adult/	3955752
4	electronic activ* track*.mp. [mp = title, abstract, original title, name of substance word, subject heading word, keyword heading word, protocol supplementary concept word, rare disease supplementary concept word, unique identifier]	0	14	adult.mp. [mp = title, abstract, original title, name of substance word, subject heading word, keyword heading word, protocol supplementary concept word, rare disease supplementary concept word, unique identifier]	4363657
5	exp Biomedical Technology/	7520	15	aged.mp. [mp = title, abstract, original title, name of substance word, subject heading word, keyword heading word, protocol supplementary concept word, rare disease supplementary concept word, unique identifier]	4057312
6	technology based.mp. [mp = title, abstract, original title, name of substance word, subject heading word, keyword heading word, protocol supplementary concept word, rare disease supplementary concept word, unique identifier]	1374	16	13 or 14 or 15	6042511
7	electronic feedback.mp.	110	17	8 and 12 and 16	42
8	1 or 2 or 3 or 4 or 5 or 6 or 7	9941	18	activity monitor.mp.	853
9	exp Exercise Therapy/ or physical activ*.mp.	87737	19	8 or 18	10780
10	exp Exercise/	119285	20	12 and 16 and 19	372

### Study Selection

Articles were screened in four steps: removing duplicates, by title, by abstract, and by full text. Once the duplicates were eliminated, articles were excluded systematically in each screening step based on the criteria listed in Table 2. Experimental studies that required participants to wear an EAMS to change their PA behavior were included in the review. Screening was conducted independently by two reviewers (ZL and JJ) and any disagreement was settled by discussion between the two reviewers. A data extraction form was completed for every study to evaluate if it met the inclusion criteria. Validation studies were eliminated under the first exclusion criterion. Studies regarding physical function or physical ability were eliminated based on the third exclusion criterion. Studies that did not include physical activity as a study variable were excluded; however, studies that only reported baseline physical activity measurements were included.

**Table 2:** Exclusion criteria

1	Not a human study population
2	Included children or adolescents
3	PA not a study variable
4	Not experimental design
5	Interventions not aimed to change behavior
6	No activity monitor device given
7	Used pedometer to change behavior
8	Not in English
9	Described a study protocol/no results reported
10	Did not meet EAMS definition

*EAMS* Electronic Activity Monitor System, *PA* physical activity

### Quality Assessment

Quality of the studies was assessed using a risk of bias tool [29] and the presence of components outlined by Khaylis et al. [12]. Assessment of bias was determined from “yes” or “no” answers to the following questions: Was the intervention length 6 months or greater? [17], were follow up measurements taken? [17], did investigators report sufficient power? [29], is the retention rate 80 % or better? [29], and were the measurements taken by a blinded assessor? [29] A “yes” answer to these questions or the presence of a Khaylis et al. component resulted in a “1” score and a “no” answer resulted in a “0” score. In the event that a grading criterion was unclear within the study, it was assumed that the feature was not present. Each article was determined to be low (score 0–4), medium (score 5–7), or high quality (score 8–10).

### Data synthesis

In an effort to synthesize the heterogeneous features of the studies, study design and result information were collected into a data abstraction form. This form consisted of information in the following areas: study design, study population, study intervention, limitations, suggested future work, and quality score (see Additional file 1).

## Results

**Fig. 1:**
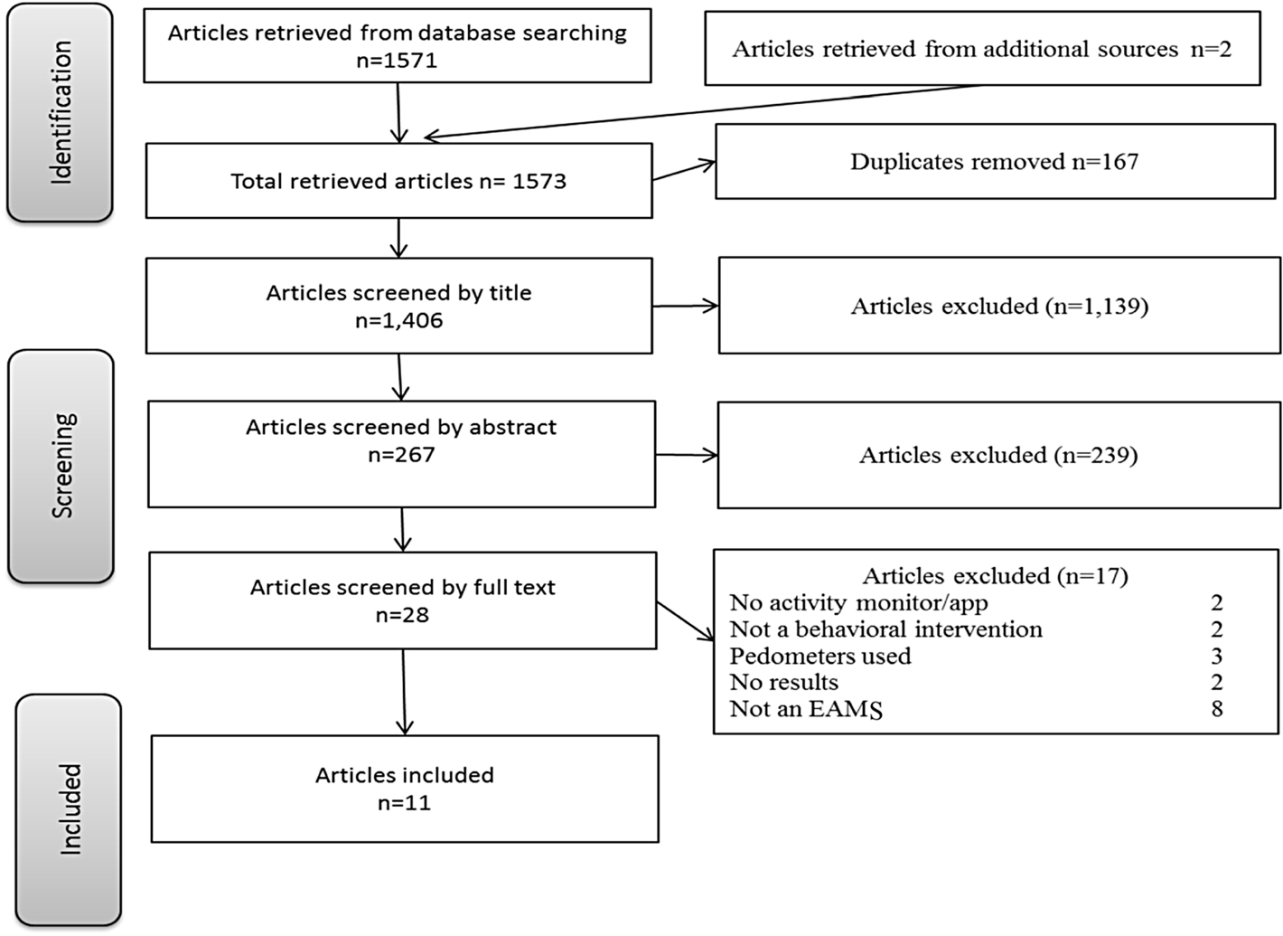
Screening process. This figure outlines the screening process. The number of articles removed at each stage are expressed in far right column

Study collection and article screening was conducted in June-July 2014. A total of 1,573 articles were retrieved from the search strategy, 167 being duplicates. Most articles were removed by screening the titles and abstracts (N = 1,378). Of the 28 remaining articles, 17 were excluded. The level of agreement between reviewers was 99 % with a moderate kappa statistic of 0.57. Fig. 1 outlines the screening process (see Additional file 2 for the complete list of excluded studies).

### Study Characteristics

The 11 studies [30–40] included 1,272 participants across five countries (Australia [30], Finland [35], the Netherlands [37, 38], UK [31, 32], and USA [33, 34, 36, 39, 40]) with a mean age ranging from 27 to 79 years. Seven studies included more than 60 % female [32–37, 39, 40], and four studies included predominantly White participants [32–34, 36]. Of the 11 studies, 10 had a randomized controlled trial design and one was a pre-experimental study [31]. Three included young (27–32 years of age) [30,37,40], five included middle-aged (40–47 years of age) [32–36], and three included aged-adults (65–80 years of age) [31, 38, 39]. Five studies had BMI inclusion criteria [32–34, 36, 39], five restricted to sedentary participants [30, 33, 34, 36, 39], three were conducted in a workplace setting [35, 37, 40], and one study restricted to chronic obstructive pulmonary disease patients [38].

The standard interventions differed across the 11 studies. Comparator interventions ranged from regular PA behavior [30, 38], providing literature on PA [35–37], wearing the EAMS but blinded to feedback [32,39,40], and standard behavioral weight loss interventions [33,34]. Four interventions were based in behavioral theory including social cognitive theory and the transtheoretical model [31, 32, 34, 36].

### Study Outcomes

All of the studies measured PA behavior. Two studies did not report PA changes: Shuger et al. [36] only reported baseline values while Thorndike et al. [40] only reported follow-up values. PA behaviors measured included: sedentary behavior (hours/day), light PA (MET-min/week), moderate PA (MET-min/week), vigorous PA (MET-min/week), leisure-time PA (MET-min/week), overall PA (MET-min/week), energy expenditure (kcals/week), walking (MET-min/week), and steps (steps/day). Five studies reported weight change [33–36, 39] and two studies reported change in body mass index [33, 36]. Table 3 outlines the study characteristics.

**Table 3:** Study design & baseline characteristics

Ref #	Type of device	Intervention group	Control group	Trial length	Subjects
[30]	GRUVE (Gruve Solution™ MUVE, Inc., USA)	online EAMS	normal daily lifestyle patterns	4 weeks	N = 33 (IG: n = 18, CG: n = 15)
					mean age (yr) IG: 29.0 ± 4.4 CG: 26.4 ± 3.0
					weight (kg) IG: 78.3 ± 20.6 CG: 77.7 ± 24.4
[31]	ActivPAL	baseline period with device-- no feedback, given consultation/education on SB and device, asked to change behavior for 2 weeks (1 week with the device)	-	24 days	N = 24
					mean age (yr) 68 ± 6
					baseline BMI(kg/cm^2^) 26.2 ± 3.7
[32]	Bluetooth Actiwatch (developed by research team),	Internet, email, and mobile phone system. The system provided feedback and motivation via email and/or text messages, and displayed activity level category	wore the EAMS device but did not receive feedback	3 months	N = 77 (IG: n = 47, CG: n = 30)
					mean age (yr) IG: 40.5 ± 7.1 CG: 40.1 ± 7.7 weight (kg) IG: 75.1 ± 11.7 CG: 73.9 ± 10.2
					BMI(kg/cm^2^) IG: 26.2 ± 2.8 CG: 26.5 ± 4.1
[33]	Sense Wear Armband (BodyMedia Fit; BodyMedia, Pittsburgh PA	TECH: behavioral lessons via mail, monthly telephone class with counselor, wore device with feedback, TECH + SBWL: behavioral lessons in person monthly (3 group, 1 in person), wore device with feedback	standard behavioral weight loss weekly meeting (3 group, 1 individual each month)	6 months	N = 51 (SBWL:N = 17, TECH + SBWL:N = 17, TECH:N = 17) mean age (yr) SBWL: 45.1 ± 9.4 SBWL + TECH: 43.3 ± 9.1 TECH: 44.1 ± 8.1weight (kg) TECH: 92.3 ± 12.1 TECH + SBWL: 102.1 ± 17.5 SBWL: 88.6 ± 12.5
					BMI(kg/cm^2^) TECH: 33.4 ± 6.3 TECH + SBWL: 34.7 ± 3.4 SBWL: 88.6 ± 12.5
[34]	Sense Wear Pro Armband (BodyMedia, Pittsburgh, PA)	INT-TECH: received SBWP, wore device weeks 1,5,9, kept paper diaries during non-tech weeks CON-TECH: received SBWP, wore device for entire trial time	SBWP: 7 in-person individualized counseling sessions (weekly in mo. 1, twice in mo. 2, once in mo. 3)	3 months	N = 57 (SBWP: N = 19, INT-TECH: N = 19, CON-TECH: N = 19)
					mean age (yr) SBWP: 40.2 ± 8.0 INT-TECH: 41.1 ± 8.3 CON-TECH: 42.6 ± 10
					weight (kg) SBWP: 89.1 ± 9.0 INT-TECH: 91.0 ± 8.8 CON-TECH: 86.6 ± 9.5
					BMI (kg/cm^2^)SBWP: 33.6 ± 2.7 INT-TECH: 33.4 ± 2.8 CON-TECH: 32.6 ± 2.7
[35]	PAM (model AM 200, PAM BV, the Netherlands)	received feedback on baseline fitness and leaflet on PA information; PAM device and distance counseling	received feedback on baseline fitness and leaflet on PA information	12- months	N = 544 (IG: N = 273, CG: N = 271)
					mean age IG: 43 ± 10 CG: 44 ± 10
					BMI(kg/cm^2^) IG: 25 ± 4 CG: 25 ± 4
[36]	Sense Wear armband (BodyMedia, Inc., Pittsburgh, PA)	GWL: 14 sessions over 4 months, 6 telephone counseling sessions over the last 5 months SWA received armband and access to web account. GWL + SWA received both GWL and SWA intervention components	received self-directed weight loss manual	9 months	N = 197 randomized (CG: N = 50, GWL: N = 49, SWA: N = 49, GWL + SWA: N = 49)
					mean age (yr) CG: 47.2 ± 8.9 GWL: 46.8 ± 12.4 SWA: 47.7 ± 11.6 GWL + SWA: 45.7 ± 10.4
					weight (kg) CG: 94.2 ± 18.2 GWL: 93.2 ± 18.9 SWA: 92.0 ± 21.0 GWA + SWA: 91.9 ± 15.7
					BMI(kg/cm^2^) CG: 33.7 ± 5.5 GWL: 33.1 ± 4.8 SWA: 33.2 ± 5.4 GWA + SWA: 33.0 ± 5.0
[37]	PAM (model AM 101, PAM BV, the Netherlands)	PAM and web-based tailored advice	single written information brochure with general PA recommendations	8 months (3 mo., 5 mo. follow-up)	N = 102 (IG: N = 51, CG: N = 51)
					mean age (yr) IG: 32.5 ± 3.4^Ɨ^ CG: 31.2 ± 3.5 weight (kg) IG: 79.0 ± 15.6 CG: 76.5 ± 13.6
					BMI(kg/cm^2^) IG: 25.9 ± 4.5 CG: 24.4 ± 3.5
[38]	MTx-W sensor (Xsens Technologies, Enschese, The Netherlands)	received activity coach (sensor and smart phone), received feedback for awareness and motivation, also received standard care	received standard care: medication and physiotherapy (group physiotherapy)	1 month	N = 34 (IG: N = 18, CG: N = 16)
					mean age (yr) IG: 65.2 ± 9.2 CG: 67.9 ± 5.7 BMI IG: 28.4 ± 7.8 CG: 29.2 ± 4.7
[39]	Fitbit (San Francisco, CA)	First 6 months received Fitbit with feedback and received counseling (face to face provided every 2 months). Last 6 months, wore Fitbit with feedback, no counseling	First 6 months wore Fitbit but didn't receive feedback or counseling. Last 6 month wore Fitbit with feedback and counseling	12 months	N = 49 (IG: N = 25, CG: N = 24)
					mean age (yr) IG: 79.1 ± 8.0 CG: 79.8 ± 6.0 weight (kg) IG: 75.7 ± 13.4 CG: 81.0 ± 13.6
[40]	Fitbit (San Francisco, CA)	given Fitbit activity monitor to wear with feedback	wore a Fitbit monitor but blinded to feedback	3 months	N = 104 (IG: N = 52, CG: N = 52)
					mean age (yr) IG: 29 ± 0.4 CG: 29 ± 0.3

^Ɨ^p < 0.05, significantly different from control

*BMI* body mass index, *CG* control group, *CON-TECH* continuous technology group, *EAMS* Electronic Activity Monitor System, *GWL* group weight loss group, *GWL + SWA* group weight loss plus SenseWear Armband group, *IG* intervention group, *INT-TECH*, intermittent technology group, *SB* sedentary behavior, *SBWL* standard behavioral weight loss group, *SBWP* standard behavioral weight control program group, *SWA* SenseWear armband alone group, *TECH* technology only group, *TECH + SBWL* technology plus standard behavioral weight loss group

### Quality assessment

The results of the quality assessment are shown in Table 4. Most of the studies were found to be of medium quality [30–35, 38, 39], two studies were low quality [37, 40], and one was of high quality [36].

**Table 4:** Quality assessment

Ref No.	[30]	[31]	[32]	[33]	[34]	[35]	[36]	[37]	[38]	[39]	[40]	No. of studies
Trial length 6 months or greater?	N	N	N	Y	N	Y	Y	N	N	Y	N	4
Were follow up assessments conducted?	N	N	N	N	N	N	N	Y	N	N	N	1
Was the study sufficiently powered?	Y	N	N	N	N	Y	Y	N	N	Y	N	4
Was the retention rate 80 % or greater?	Y	Y	Y	N	Y	N	N	N	Y	Y	Y	7
Was the assessor blinded?	N	N	N	N	N	N	Y	N	N	N	N	1
Did participants self-monitor?	Y	Y	Y	Y	Y	Y	Y	Y	Y	Y	Y	11
Did participants receive feedback?	Y	Y	Y	Y	Y	Y	Y	Y	Y	Y	Y	11
Was there social support?	N	N	Y	Y	N	N	Y	N	Y	N	N	4
Was there a structured behavioral program?	N	Y	Y	Y	Y	N	Y	N	N	Y	N	6
Individually tailored program?	Y	Y	Y	Y	Y	Y	Y	Y	Y	Y	Y	11
Total quality	MEDIUM	MEDIUM	MEDIUM	MEDIUM	MEDIUM	MEDIUM	HIGH	LOW	MEDIUM	MEDIUM	LOW	

### EAMS features

Gruve (Gruve Solution™ MUVE, Inc., USA) [30], activPAL (PAL Technologies Ltd., Glasgow, Scotland) [31], Bluetooth Actiwatch [32], Sense Wear armband (BodyMedia, Pittsburgh PA) [33, 34, 36], PAM (model AM 200/model AM 101, PAM BV, the Netherlands) [35, 37], MTx-W sensor (Xsens Technologies, Enschese, The Netherlands) [38], and Fitbit (Fitbit, San Francisco, CA) [39, 40] devices were used in the studies. Gruve, PAM, and Fitbit are commercially available for individual use while the other EAMSs are available through distributors. The devices were worn on different parts of the body according to the monitor instructions. Locations included: along the iliac crest [30, 37–39], upper thigh [31] or upper arm [33, 34, 36]. All of the EAMSs allowed for self-monitoring and individualized feedback. Research accelerometers were manipulated by the investigator to provide automated self-monitoring to the wearer [31, 33, 34, 36]. Feedback from the EAMS was administered daily [31–34, 36, 38] or weekly [30, 35, 37]. The feedback was delivered through the device [31, 32], via text messages [38], emails [30], or online monitoring system [30, 33–37].

### PA and weight change

The study design and the change in study outcomes are outlined in Table 3 and Table 5, respectively.

**Table 5:** Mean change (effect size over time) in study outcomes

Ref #	Weight (kg)	BMI (kg/cm^2^)	SB (hours/day)	LPA (MET-min/week)	MPA (MET-min/week)	VPA (MET-min/week)	LTPA (MET-min/week)	Overall (MET-min/week	EE (kcals/week)	Walking (MET-min/week)	Steps (per day)
[30]	-	-	IG: −2.3*	IG: 2.5*	IG: 455*	IG: 442*	-	-	-	IG: 1057*	-
			(−1.28)	(1.35)	(1.26)	(0.67)				(1.95)	
			CG: 0.5	CG: −	CG: 12	CG: 20				CG: −54	
			(0.25)	0.4 (−0.22)	(0.06)	(0.07)				(−0.23)	
			*d: −1.63*	*d:2.12*	*d: 1.28*	*d:0.96*				*d: 3.13*	
[31]	-	-	0.98*	-	-	-	-	-	-	-	1193*
			(−0.27)								(0.18)
[32]^a^	-	-	IG: −5.9^Ɨ^	-	-	-	IG: 4.1^Ɨ^	IG: 12	-	-	-
			CG: 1.4				CG: −5.5	CG: 4.0			
			*d: −3.11*				*d: 3.15*	*d: 2.22*			
[33]	TECH_1_:	TECH_1_:	-	-	-	-	-	-	TECH_1_:	-	-
	−5.9*	−2.1							1066.2*		
	(−0.46)	(−0.52)							(1.20)		
	TECH+	TECH+							TECH+		
	SBWL_2_:	SBWL_2_:							SBWL_2_:		
	−8.8*	−3.1							713.9*		
	(−0.50)	(−0.91)							(0.93)		
	SBWL:-3.7*	SBWL: −1.4							SBWL: 473.9*		
	(−0.29)	(−0.38)							(0.691)		
	*d*_*1*_ *: 0.11*	*d*_*1*_ *: −0.10*							*d*_*1*_ *: 0.44*		
	*d*_*2*_ *: 0.55*	*d*_*2*_ *: −0.03*							*d*_*2*_ *: 0.61*		
[34]^a^	SBWP:4.1* INT-TECH_1_: 3.4* CON-TECH_2_:6.2^Ɨ^	-	-	-	-	-	SBWP: 281*INT-TECH_1_: 1286.7* CON-TECH_2_: 1112.3	-			
	*d*_*1*_ *:-0.23*						*d*_*1*_ *:0.46*				
	*d*_*2*_ *:0.62*						*d*_*2*_ *:0.74*				
[35]	IG: 1 CG: 0^a^ *d: −0.08*	-	-	-	-	-	-	6 mo_1_ IG: −88			
								(−0.06)			
								CG:185			
								(0.11)			
								12mo_2_			
								IG:-36			
								(−0.02) CG: 80			
								(0.05)			
								*d*_*1*_ *:-0.24*			
								*d*_*2*_ *: −0.17*			
[36]	4 months	4 months	-	-	-	-	-	-	-	-	-
	CG: −0.99	CG:-0.4									
	(−0.33)	(−0.43)									
	GWL_1_: −1.1	GWL_1_: −0.33									
	(−0.37)	(−0.36)									
	SWA_2_:	SWA_2_:									
	−2.67*	−0.9									
	(−0.90)	(−0.99)									
	GWA+	GWA+									
	SWA_3_:	SWA_3_:									
	−3.49*	−1.26									
	(−1.17)	(−1.38)									
	*d*_*1*_ *:-0.16*	*d*_*1*_ *:0.10*									
	*d*_*2*_ *:-0.92*	*d*_*2*_ *: −0.32*									
	*d*_*3*_ *:-1.46*	*d*_*3*_ *:-1.08*									
	9 months CG:-1.1	9 months CG: −0.36									
	(−0.30)	(−0.39)									
	GWL_1_:	GWL_1_:									
	−1.86*	−0.7									
	(−0.63)	(−0.77)									
	SWA_2_:	SWA_2_:									
	−3.55*	−1.17									
	(−1.20)	(−1.29)									
	GWA+	GWA+									
	SWA_3_:	SWA_3_:									
	−6.59*^Ɨ^	−2.28									
	(−2.21)	(−2.49)									
	*d*_*1*_ *:-0.44*	*d*_*1*_ *:-0.34*									
	*d*_*2*_ *:-1.23*	*d*_*2*_ *:-0.65*									
	*d*_*3*_ *:-2.51*	*d*_*3*_ *:-2.20*									
[37]^b^	-	-	3 months IG:10 CG: −905 8 months IG: −465 CG: −33 (min/week)	3 months IG: 6 CG: −42 8 months IG: −130 CG: −127 (min/week)	3 months IG: −15 CG:-30 8 months IG:30 CG:-30 (min/week)	3 months IG: −90 CG: −7 8 months IG: −50 CG: −5 (min/week)	-	-	-	-	-
[38]^a^	-	-	-	-	-	-	-	-	-	-	IG: −163 CG: −639 *d: 1.08*
[39]^b^	IG: −1.01*	-	-	-	-	-	-	IG: −217.8 activity counts CG: −583.68 activity counts	-	-	-
	CG:-0.99*										
[40]^c^	-	-	-	-	-	-	-	-	-	-	IG: 5967 CG: 5341

*p < 0.05, significant within group change; ^Ɨ^p < 0.05, significantly different from control

*d:* effect size (Cohen’s d) between experimental group and control group; d value of 0.8 or greater indicates a large effect size

^a^:effect size over time could not be calculated; ^b^:reported outcome median (IQR), no effect size calculated; ^c^: actual values, no differences reported

*BMI* body mass index, *CG* control group, *CON-TECH* continuous technology group, *EAMS* Electronic Activity Monitor System, *EE* energy expenditure, *GWL* group weight loss group, *GWL + SWA* group weight loss plus SenseWear Armband group, *IG* intervention group, *INT-TECH* intermittent technology group, *LPA* light physical activity, *LTPA* leisure-time physical activity, *MPA* moderate physical activity, *SB* sedentary behavior, *SBWL* standard behavioral weight loss group, *SBWP* standard behavioral weight control program group, *SWA* SenseWear armband alone group, *TECH* technology only group, *TECH + SBWL* technology plus standard behavioral weight loss group, *VPA* vigorous physical activity

Of the 11 studies, 9 measured changes in PA [30–35, 37–39]. Of these, 5 reported significant pre-post intervention differences [30–34], and 1 reported a significant increase compared to the control group [32]. Increases in PA ranged from 2.5 to 1,286 MET-min/week and 473 to 1066 kcals/week. The effect size for change in PA pre-post difference ranged from −0.22 to 1.9 while the effect size compared to the control group ranged from −0.24 to 3.15. Four studies reported change in sedentary behavior [30–32, 35]. Of these, 3 reported significant pre-post improvement [30–32], and Hurling et al. reported significant decrease compared to the control group [32]. Decreases in sedentary time ranged from −1 to −905 hours/day. The effect size for pre-post change in sedentary behavior ranged from −1.28 to 0.25 while the effect size compared to the control group ranged from −3.12 to −1.63.

Of the 5 studies that measured changes in weight, 4 reported significant decreases over time [33, 34, 36, 39], and 2 reported significant differences between the EAMS group and another group [34,36]. Of these studies, three monitored dietary intake [33,34,36]. Weight loss among all study groups ranged between −0.99 and −8.8 kg. The effect size for pre-post intervention change in weight ranged from −2.21 to −0.30 while the effect size compared to the control group ranged from −2.51 to 0.62.

In the three studies that found significant differences between the EAMS condition and another on physical activity or weight outcomes, the other conditions were wearing the EAMS but blinded to feedback [32], intermittent wear of an EAMS combined with a standard behavior weight loss program [34], and receiving a self-directed weight loss manual [36].

### Intervention feasibility

Feasibility of the EAMS intervention was evaluated from the retention rate within the study period and compliance. Of the 11 studies, 7 studies reported a retention rate of 80 % or better [30–32, 34, 38–40]. Among the interventions that had greater than a 20 % attrition rate, 2 studies saw higher retention rate in the intervention group [33, 36]. Only 7 studies reported on a measure of compliance, either time the device was worn or the frequency of using the EAMS system [31–35, 37, 38]. More than 80 % of participants met recommended wear time [31, 32, 38]. The time the device was worn ranged from 16.2-17.4 hours/day [33] and 63.8-71 hours/week [34]. Utilization of the online EAMS ranged from 0.6 [35] to 0.9 times per week [37]. There was significant correlation between the time the device was worn and change in body weight [34] as well as change in activity [38].

## Discussion

This systematic review summarizes the results of EAMS interventions aimed to change PA behavior available by August 2014 [30–40]. The results suggest that EAMS may encourage improvements in PA and weight loss, which is comparable to other technology-based interventions [12,20]. However, data on their effectiveness compared to other interventions and standard of care is equivocal.

### Physical activity

The heterogeneity in reporting PA makes it difficult to compare PA changes across studies. PA was measured in MET-min/week, kcals/week, steps per day, and activity counts per day. Time spent in PA was further stratified into light, moderate, vigorous intensity, leisure time and walking. Better uniformity in reporting PA behavior would improve attempts at aggregation and synthesis.

Two studies were deemed low quality with a high risk of bias [37, 40]; therefore their results should be reviewed with skepticism. Of the 8 studies of medium quality that report end of study PA, 5 studies showed significant PA improvements [30–34] and 3 studies found significant sedentary behavior improvements [30–32] in the EAMS group. One study [32] found significant difference in PA and sedentary behavior between the EAMS group and the study control group [30].

We hypothesize five possible reasons why the other studies did not find significant results: they were not sufficiently powered, did not include all of the Khaylis et al. [12] components, had a largely active population at baseline, the comparator group received a high intensity intervention and/or some EAMS may not produce statistically different change in PA.

We cannot definitively evaluate why some studies found significant results, but we hypothesize three possible reasons. Of the five studies that found a significant difference from pre to post intervention, three [31, 32, 34] were grounded in behavioral theory. EAMSs may be more appropriate for implementation as a component within a larger behavioral intervention, rather than as an intervention in and of themselves. Some of the EAMS used within these interventions [32–34] were research grade devices which may have implemented more BCTs and provided richer feedback on behavior than devices marketed for individual use such as Fitbit. The single study that found significant differences between groups for physical activity [32] utilized a true control group that did not receive any education or counseling. The studies without significant differences utilized more active alternative interventions. Trials with innovative study designs, such as Multiphase Optimization Strategy trials, would be well-suited to investigating the effects of nesting EAMSs within larger interventions, comparison of different devices, and the intensity of comparator interventions.

### Weight loss

Five studies included in this review reported change in body weight [33–36, 39]. Two of these also reported change in body mass index [33, 36]. Of these 5 studies, 4 reported significant decreases over time [33, 34, 36, 39] and 2 found significant difference between the intervention and comparator group [34, 36]. Both studies that found significant group differences incorporated dietary modifications into the intervention [34,36]. There was no significant change in body mass index. All but one of the studies was of medium quality [33–35, 39]. Shuger et al. was of high quality [36]. We present six hypotheses as to why significant weight loss was not observed in all studies: studies were not sufficiently powered, no dietary restrictions [41], short intervention time, not a severely obese population [5], the comparator group received a high intensity intervention and/or some EAMS may not produce statistically different change in weight. The proposed hypotheses presented in the previous physical activity section likely also apply to weight outcomes.

### Cost Effectiveness

Interventions that are technology based can be more cost effective than face-to-face interventions [7]. To our knowledge, the study conducted by Shuger et al. is the only study that had subsequent economic analysis. The analysis found that the Sense Wear alone group was the most cost effective with $51/participant for each kg lost followed by the group weight loss and Sense Wear group ($55/participant/kg lost) and the group weight loss group ($129/participant/kg lost) [42]. More research is needed to evaluate the cost-effectiveness of the studies included in this review and future EAMS interventions. Due to the potential cost-effectiveness, EAMS appear to be a promising tool for broad dissemination of behavioral intervention components.

### Limitations of studies

There are several limitations to the studies included in this review. Only five studies had weight as an outcome from the change in PA behavior. Few studies obtained follow-up analysis to investigate maintenance and most interventions included a predominant population of White women.

Only four studies [31, 36, 38, 39] used an objective measurement of PA. These studies used both the EAMS device [31, 36] and other devices [38, 39]. Validation studies have shown that some EAMS can produce valid measures of energy expenditure [43–47]. Objective measures could be provided by the EAMS themselves in an effort to reduce recall bias.

The studies did not meet CONSORT reporting guidelines. Only three studies were based in behavioral theory however all EAMS devices encourage behavioral change. Thus, the studies should specify their interventions based on the BCT taxonomy v1 that meets CONSORT reporting guidelines [48].

Only one study [36] reported a blind assessor. Some studies did not report on secondary outcomes if they were not significant, subjecting to reporting bias. This limits the comprehensiveness of this review and our understanding of an EAMS within an intervention. The randomized controlled trial design and a large volunteer population limits the studies’ generalizability to a relatively healthy subgroup of the population.

### The state of the literature and recommendations for future work

Behavioral physical activity interventions have progressed from face to face interventions with paper diaries for self-monitoring to using technology to facilitate self-monitoring. Interventions that use Internet diaries, pedometers, and handheld personal digital assistants have found greater weight loss compared to the traditional paper diaries [12]. Furthermore, Internet-based physical activity interventions are effective compared to wait-list controls [49]. Technology shows promise as an intervention modality but it is not without its limitations. Some technologies only provide a modest effect [16] and there is insufficient evidence that technology-based interventions are more effective than traditional face-to-face behavioral interventions [50]. The present review advances the literature of physical activity interventions by evaluating EAMS that implement BCTs and highlighting important future directions for inquiry.

This review led to several conclusions about gaps in the literature and what is needed from future work within EAMS research. EAMS research requires special considerations in study design and reporting. We have grouped these suggestions into several major recommendations:

1. Authors should explicitly discuss EAMS versions and the BCTs implemented in those versions. In a recent study, we found that within several months, multiple EAMS had increased the number of BCTs they included substantively, requiring re-coding [25]. Based on that study and prior use of the EAMS reviewed here, we suspect that later studies using more sophisticated EAMS may produce more positive results due to better implementation of BCTs. Better description of the techniques included will assist greatly in comparing results across studies and interpreting the literature.
2. The rationale for EAMS use should be clear and based on evidence and behavior change theory. Hypothesized mechanisms of change should be emphasized and measured rigorously to allow for tests of mediation. It is reasonable to hypothesize that EAMS may be equivalent to standard face-to-face interventions, more effective due to their ability to improve upon delivery of various BCTs or ease of use/enjoyment, or less effective due to difficulty of use or poor engagement. The studies reviewed here seemed to have disparate rationales for why EAMS might work, which might have contributed to the heterogeneity and difficulty of interpreting results. Measurement of intermediate variables such as autonomous motivation, self-efficacy, goal-setting and planning will provide valuable information whether the behavioral/health outcome results are significant or not. Beyond whether or not EAMS work, we must know why or why not they work. Without stronger conceptual models and study designs, comparisons across studies will remain difficult.
3. If EAMS are to be used, their potential should be maximized. That is, the BCTs they are capable of implementing should all be available to participants. For example, some EAMS can provide social support and social comparison which are major mechanisms by which EAMS are hypothesized to affect PA. None of the included studies reported utilizing these features. The body of EAMS research will be more rigorous if researchers maximized interventions by providing all BCTs.
4. Process evaluations on indicators of participant engagement are necessary to further understand how and why PA may change (or not change) while using an EAMS. Many of these systems allow investigators to “friend” participants and conduct daily surveillance of their app use. App usage (i.e., how often they checked their steps per day), engagement with others (i.e., friending other people, commenting on others’ progress), and engagement with additional app functions (i.e., monitoring other health indicators, creating smart alarms, turning on notifications) may provide richer information on the extent to which participants truly used the EAMS.
5. An EAMS provides individualized recommendations, therefore the EAMS provided should be individualized to an individual’s preference. The evaluation of desired EAMS features by a population of individuals is a necessary area of research.

In addition to addressing the previous recommendations, more rigorous research is needed to improve the quality of the research being conducted. Future studies should focus on being sufficiently powered, conducting follow up assessments, objectively measuring PA, including a diverse population, and completing analyses of cost effectiveness and public health impact (e.g., RE-AIM) [51]. Efforts to report outcomes using similar units for PA and behavioral change strategies would facilitate a meta-analysis, which would be able to make conclusions that this qualitative review could not. The most interpretable PA units are METs-min/week and steps per day while the behavioral change strategies reported should use the CALO-RE taxonomy [14].

### Strength and Limitations of review

This review is limited in its scope of literature and understanding. Only EAMS interventions were included in this review and not all interventions that utilize self-monitoring technology. There are other reviews available that include electronic self-monitoring [10], all activity monitors [21], mobile phone technologies [16,17], and other innovative technologies [7,52]. Some EAMS studies may not have been captured and ongoing EAMS studies were not evaluated, despite rigorous search efforts. EAMS technology changes quickly and frequently, the devices discussed contained fewer BCTs than versions available at a later date [25]. Due to the heterogeneity of study design and PA outcomes it is difficult to compare and contrast between studies. For this reason, EAMS efficacy was limited to a qualitative review.

To our knowledge, this is the first systematic review on EAMSs. The clear definition of an EAMS described in this review is a major strength. This review also follows a thorough, systematic methodology. This review also summaries the current evidence within this emerging field of research as well as informs the design and reporting of studies in the future and those currently underway [53].

## Conclusions

EAMS technology is readily available and utilized commercially by health professionals to motivate patients [7, 22, 26, 27]. The EAMS interventions studied in this review demonstrated ability to increase PA and decrease weight. Though comparisons to other interventions produced equivocal results, effect sizes suggest potentially clinically significant outcomes [54]. The heterogeneity and reported limitations of the studies suggest that more research is needed. Future studies should be well-designed, high quality randomized controlled trials that would facilitate a meta-analysis, evaluate which EAMS features are relevant to participants, and test the hypotheses presented in this discussion.

## Additional files

Additional file 1:
**Data abstraction form.** This file provides further details of each study included in this review. Additional file 2:
**Excluded studies.** This file provides a list of all the studies that were captured from database search but did not meet the inclusion criteria.

## Abbreviations

BCT: Behavioral Change Techniques
EAMS: Electronic Activity Monitor System
PA: Physical Activity
